# Chagas Disease in the Non-Endemic Area of Rome, Italy: Ten Years of Experience and a Brief Overview

**DOI:** 10.3390/idr16040050

**Published:** 2024-07-24

**Authors:** Maria Letizia Giancola, Andrea Angheben, Laura Scorzolini, Stefania Carrara, Ada Petrone, Antonella Vulcano, Raffaella Lionetti, Angela Corpolongo, Rosalia Marrone, Francesca Faraglia, Tommaso Ascoli Bartoli, Patrizia De Marco, Maria Virginia Tomassi, Carla Fontana, Emanuele Nicastri

**Affiliations:** 1 Clinical Department, National Institute for Infectious Diseases “Lazzaro Spallanzani”, Istituto di Ricovero e Cura a Carattere Scientifico (IRCCS), 00149 Rome, Italy; angela.corpolongo@inmi.it (A.C.); francesca.faraglia@inmi.it (F.F.); tommaso.ascoli@inmi.it (T.A.B.); patrizia.demarco@inmi.it (P.D.M.); mvirginia.tomassi@inmi.it (M.V.T.); emanuele.nicastri@inmi.it (E.N.); 2 Department of Infectious—Tropical Diseases and Microbiology, Istituto di Ricovero e Cura a Carattere Scientifico (IRCSS) Sacro Cuore Hospital, Negrar, 37024 Verona, Italy; andrea.angheben@sacrocuore.it; 3 Laboratory of Microbiology and Biological Bank, National Institute for Infectious Diseases “Lazzaro Spallanzani”, Istituto di Ricovero e Cura a Carattere Scientifico (IRCCS), 00149 Rome, Italy; stefania.carrara@inmi.it (S.C.); antonella.vulcano@inmi.it (A.V.); carla.fontana@inmi.it (C.F.); 4 Radiology Unit, National Institute for Infectious Diseases “Lazzaro Spallanzani”, Istituto di Ricovero e Cura a Carattere Scientifico (IRCCS), 00149 Rome, Italy; ada.petrone@inmi.it; 5 Infectious Diseases and Epatology, Transplant Department, National Institute for Infectious Diseases “Lazzaro Spallanzani”, Istituto di Ricovero e Cura a Carattere Scientifico (IRCCS), 00149 Rome, Italy; raffaella.lionetti@inmi.it; 6 National Institute for Health, Migration and Poverty (INMP), 00153 Rome, Italy; rosalia.marrone@inmp.it

**Keywords:** Chagas disease, *Trypanosoma cruzi*, non-endemic country, benznidazole, screening

## Abstract

Chagas disease (CD) is a parasitic infection endemic in Latin America and also affects patients in Western countries due to migration flows. This has a significant impact on health services worldwide due to its high morbidity and mortality burden. This paper aims to share our experience at the National Institute for Infectious Diseases “Lazzaro Spallanzani”, IRCCS, in Rome, Italy, where to date, a total of 47 patients—mainly Bolivian women—diagnosed with CD have received treatment with benznidazole, with all but one presenting with chronic disease. Most of the patients were recruited through the first extensive screening program held in 2014 at our Institute. About a quarter of our patients showed adverse effects to benznidazole, including a case of severe drug-induced liver injury, but 83% completed a full course of treatment. In addition to the description of our cohort, the paper reports a brief overview of the disease compiled through a review of the existing literature on CD in non-endemic countries. The growing prevalence of CD in Western countries highlights the importance of screening at-risk populations and urges public concern and medical awareness about this neglected tropical disease. There are still many unanswered questions that need to be addressed to develop a personalized approach in treating patients.

## 1. Introduction

The Chagas disease (CD) is a parasitic infection caused by *Trypanosoma cruzi* (*T. cruzi*) and is endemic in 21 Latin American countries. However, due to migration flows, the multiple routes of transmission and its chronic nature, this disease also affects patients in Western countries, where it is poorly known by patients, and physicians too, and is rarely recognized. Consequently, it has a significant impact on health services not only in endemic countries but also worldwide due to its high burden of morbidity and mortality [1]. 

The World Health Organization (WHO) estimates that 6 million people live with CD worldwide [2]. Some issues, such as accessibility to health services and treatment for those affected, are specific to this disorder and make patient care more challenging than for other diseases.

This paper aims to report our experience at the National Institute for Infectious Diseases “Lazzaro Spallanzani”, IRCCS, in Rome, Italy, where, to date, a total of 47 patients diagnosed with CD, mainly women originally from Bolivia, have received treatment with benznidazole. In addition, we reviewed the existing literature to create a brief but comprehensive overview on CD from the perspective of non-endemic countries.

### 1.1. Overview on CD

#### 1.1.1. Etiology and Pathogenesis

*T. cruzi* is a hemoflagellate protozoan parasite that causes CD. It was identified as the etiological agent of CD over 100 years ago by Sir Carlos Justiniano Ribeiro Chagas. The parasite has high genetic and phenotypic diversity and has been classified into six discrete typing units. *T. cruzi* infection has three cycles: domestic, peridomestic, and wild. These cycles involve reservoirs, vectors (bugs), and humans, and the parasite is transmitted by blood-sucking triatominae, also known as kissing bugs (*Triatoma*, *Panstrongylus*, *Rhodnius*), which are widely distributed in both forested and drier areas of Latin America. The parasites are excreted with feces in the form of trypomastigotes and can enter the body through skin wounds or mucosal membranes contact, especially the conjunctiva, close to the site of the insect bite.

**Figure 1:**
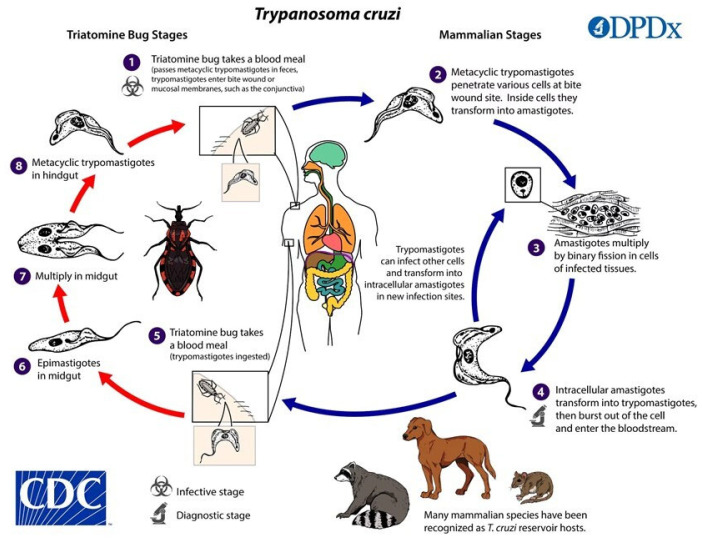
*Trypanosoma cruzi* life cycle (https://www.cdc.gov/dpdx/trypanosomiasisamerican/index.html, accessed on 2 February 2024).

The parasites then continue their life cycle, enter the circulatory system, and become a source for the blood meal of the triatomine bugs again [3,4] (Figure 1).

In some cases, transmission can occur by consuming food or drink contaminated with triatomine faeces [5]. 

Vector control has successfully interrupted transmission in certain areas of Central and South America [4]. However, *T. cruzi* can also be transmitted through means other than vectors, which are the main modes of transmission in non-endemic countries. These alternative routes include blood transfusion, organ and tissue transplantation, as well as congenital transmission. The rate of mother-to-child transmission is estimated to be around 4.7% [6].

#### 1.1.2. Epidemiology

CD was originally confined to inland rural areas of Latin American countries, but due to migration, it has now spread to several countries worldwide. In Latin America, *T. cruzi* infection is most prevalent in Bolivia (6.1 cases per 100 inhabitants), Argentina (3.6), Paraguay (2.1), Ecuador (1.4), El Salvador (1.3), and Guatemala (1.2) [7]. In non-endemic countries, cases have been reported in the United States, Europe, Australia, and Japan [8]. In the US, CD is a significant cause of parasitic infection, with an estimated number of cases of between 240,000 and 350,000 [9,10].

The prevalence of CD among Latin American migrants living in Europe is not negligible.

A systematic review and meta-analysis of 18 studies conducted in five European countries found a random effect pooled prevalence of 4.2% (95%CI: 2.2–6.7%). However, there was high heterogeneity in the prevalence of CD among the studies. Migrants from Bolivia had the highest prevalence of CD at 18.1% (95%CI: 13.9–22.7%), followed by Paraguay [11]. Basile et al. reported an annual incidence rate in Europe of congenital transmission between 0 and 3 cases per 1000 pregnancies in women from endemic countries. However, in the same study, 94–96% of *T. cruzi* infections are estimated to be underdiagnosed [12]. 

The proportion of CD in newborns of Latin American mothers in Italy is estimated to be 1:1670 [13]. Italy has a significant burden of CD, ranking third after the US and Spain among non-endemic countries [12]. In Southern Europe, Italy, along with Spain and Portugal, is actually the most affected by migration flows from Latin America.

A recent study estimates that there are 3268–5015 individuals in Italy infected with *T. cruzi*: with the majority of cases located in Lombardy, Latium, Piedmont, and Tuscany [14]. 

During a 5-month screening program offered at our Institute to migrants from Latin American countries residing in Rome, Italy, we found a seroprevalence of 8.7% (32 out 368 participants). Among Bolivian migrants, the positive serology for *T. cruzi* reached 23.5%, while 6.2% of positive persons were born in El Salvador, and 3.1% came from each of the following countries: Brazil, Colombia, and Ecuador [15].

#### 1.1.3. Clinical Manifestation

From a clinical viewpoint, CD has an acute and a chronic phase. After transmission by a vector, the incubation period is 1–2 weeks. In the acute phase, the parasite is detectable in the blood. Symptoms are often absent or mild and non-specific, such as fever, malaise, generalized swelling, lymph node enlargement, and enlargement of the liver and spleen. In rare cases, a distinctive sign called the Romaña sign—which is unilateral palpebral oedema—can be observed [4].

Severe meningoencephalitis or myocarditis may occur in less than 1% of acute cases. Within 2–3 months the parasite level in the blood decreases, and the chronic phase begins. Most infected individuals do not show any symptoms during the chronic phase for their entire life, and therefore, they remain in the so-called indeterminate chronic phase, while 20–40% of the infected individuals are estimated to show progression to organ damage in the decades following the disease acquisition. In this chronic determinate phase of the disease, mainly the heart; the digestive system; and, less commonly, the nervous system are affected [3,4]. 

Chronic Chagas cardiomyopathy mainly affects the heart’s conduction-system, leading to various heart rhythm abnormalities, right bundle-branch block or left anterior fascicular block, bradycardia, atrial fibrillation or flutter, atrioventricular blocks, and ventricular tachycardia. Cardiac involvement can progress to dilated cardiomyopathy and congestive heart failure. Patients may develop aneurysms or atrial thrombosis, leading to strokes or other thromboembolic events, or sudden death [16,17]. Although the pathogenesis of Chagas cardiomyopathy is not completely understood, the persistence of the parasite and the host immune response are considered crucial factors. Gastrointestinal CD is less common. It is based on the damage of intramural neurons and mainly affects the esophagus, the colon or both, causing motility disorders or megaviscera (megaesophagus, megacolon or both). Symptoms include dysphagia, odynophagia, esophageal reflux, regurgitation, aspiration, cough, and weight loss in case of esophagus involvement. Megacolon is characterized by prolonged constipation and may give rise to fecaloma, volvulus, and bowel ischemia [18,19]. 

In the chronic phase, if patients with positive *T. cruzi* antibodies have no symptoms or signs and no evidence of organic cardiac and intestinal lesions on ECG, chest X ray, and on any other radiological imaging or endoscopic study, the disease is classified as “indeterminate”. 

In immunocompromised hosts—such as those with advanced HIV infection, organ transplant recipients, or those taking immunosuppressive drugs—reactivation of chronic *T. cruzi* infection can occur, leading to high mortality rates. Patients with both HIV infection and low CD4 cell counts may experience reactivation in up to 20% of cases, and may present most commonly with meningoencephalitis, brain abscesses or both, and less frequently with acute myocarditis [3,4].

#### 1.1.4. Diagnosis

During the acute phase, which lasts 8–12 weeks, a positive blood smear, direct microscopic observation after concentration (Strout technique for adults or Microstrout for newborns), blood culture, or polymerase chain reaction (PCR) can confirm the diagnosis, while serology is less useful. Unfortunately, most acute infections are not suspected nor detected. The indeterminate chronic phase of the infection can be diagnosed through the detection of antibodies (IgG); PCR may show varying positivity during this stage due to extremely low and intermittent parasitemia. Congenital infection is diagnosed by a positive blood smear (or, currently, PCR) on the newborn’s blood within the first weeks of life or by the presence of IgG in serological tests at 9 months of age or later once maternal IgG is lost. 

Serological methods are based on detecting IgG antibodies against *T. cruzi* antigens in the serum. Various serological tests are available, including conventional tests (based on antigens obtained from the whole parasite) and unconventional tests (based on recombinant antigens). The World Health Organization (WHO) recommends that the diagnosis of chronic CD should be based on two positive tests obtained by two different methods based on different antigens, such as a conventional test followed by an unconventional test [20,21]. Sometimes, results are discordant (one method tests positive and the other negative) [15,22], which complicates the diagnostic process and reinforces the challenge of a certain diagnosis, which is of great importance in clinical practice. A third confirmatory test is therefore mandatory. Serological tests are typically performed in non-endemic countries in reference laboratories, and access to the tests can be a challenge in non-endemic areas [23].

#### 1.1.5. Screening Tests

Screening for chronic CD plays a crucial role in identifying asymptomatic individuals with unknown chronic infections. Asymptomatic patients from endemic areas should always undergo serological examination. Extensive screening of at-risk populations helps to find individuals with chronic infections, which are the majority, for whom anti-parasitic therapy can be prescribed to reduce the risk of complications and interrupt CD transmission, such as from mother to child [24]. It is essential to identify the target population for screening, including individuals with epidemiological risk factors for *T. cruzi* infection, family members of persons diagnosed with CD, and individuals born or having lived for an extended period (more than 6 months) in endemic countries [10,21,25]. Additionally, screening is recommended for donors from endemic countries and organ and tissue recipients at risk of reactivation, infants and children born to infected and untreated mothers, girls before their childbearing age, and pregnant women given the great benefit of the treatment in interrupting maternal–fetal transmission or early detection of congenital infection [10,21,25,26,27].

In Italy, screening for anti-*T. cruzi* was introduced in 2015 for blood donations [28] and in 2017 for organ donations [29]. The introduction of the screening of pregnant women at risk is under discussion (Angheben A, personal communication).

#### 1.1.6. Clinical and Radiological Assessment and Staging

In the clinical evaluation of chronic CD, it is important to carefully note the signs and symptoms presented by patients, but the instrumental investigation for subclinical or silent complications is also very useful.

Once the diagnosis of CD has been established by a confirmed positive serological result, cardiac, gastrointestinal, and sometimes neurological involvement should be investigated. 

##### Cardiological Assessment

A clinical evaluation of cardiovascular or digestive signs and symptoms is required, followed by ECG and echocardiography to further investigate and detect signs of silent cardiac involvement. Chagas cardiomyopathy, arrhythmias, left ventricular dysfunction, and congestive heart failure should be investigated. In some patients, 24 h Holter monitoring is indicated; exercise testing and MRI may be useful in selected cases. ECG abnormalities that are considered to be a sign of Chagas heart disease are complete right bundle branch block and left anterior fascicular block; complex ventricular arrhythmias and sustained ventricular tachycardia; supraventricular tachyarrhythmias (atrial fibrillation, atrial flutter and atrial tachycardia); type II and complete second-degree atrio-ventricular blocks; sinus bradycardia < 50 beats per minute; and presence of a permanent pacemaker [30,31].

Changes or new ECG abnormalities during clinical follow-up are the most important signs given the continued risk factor for CD patients to develop cardiac problems. Indeed, the presence of the parasite in cardiac tissue could persist, and a negative PCR for *T. cruzi* in the blood does not rule out the presence of the parasite in the tissues, even in treated patients, as the efficacy of the treatment is not absolute. In these cases, ECG changes can be indicative of disease progression. In addition, ECG in follow-up is useful because some of the abnormal findings that could be related to chronic CD are common in the seronegative population of the same age group, and longitudinal observation can clarify the evolution of the disease [30,31].

Conventional radiology examinations based on X-rays are the standard method for staging the patient and investigating possible organ damage.

The chest X-ray (postero-anterior and lateral projection), which is easy and quick to perform, should be carried out in all patients to assess the cardiac silhouette. It provides information of the cardio-mediastinal index and may reveal the presence of cardiomegaly. Echocardiography better assesses heart function. The Brazilian Consensus Classification classifies the patients into five groups based on severity of heart failure [32]. 

The classification suggested by Andrade is the most commonly used to evaluate the cardiac involvement [33]. It includes four categories combining the results of serology, ECG, and chest X-ray or echocardiogram.

##### Gastrointestinal Assessment

X-ray with contrast of esophagus and enema are used to investigate gastrointestinal damage, which may be of varying degrees, up to dilatation of the esophagus (four degrees of involvement up to megaesophagus), colon (megacolon) or both. In patients with suspected chagasic digestive disease, Rezende’s and Ximenes’s techniques for barium contrastographic study are the standard [34,35]. These examinations allow the visualization of the shape of the viscera and the analysis of their diameter and wall contours and also the kinetic activity of the esophagus.

Opaque enema, mostly using barium, is the gold standard for the diagnosis of megacolon, showing dilation or stretching or the presence of fecaloma. Although megaesophagus and megacolon have been recognized as related to the clinical picture of CD, there are doubts on the pathological significance of a solitary dolichocolon. Esophageal manometry can be useful for differential diagnosis and adds functional information.

Ultrasound of the abdomen is not required for staging but can be considered a complementary examination as CD patients frequently present with other findings, e.g., gallstones. 

Computed tomography and magnetic resonance imaging are more sensitive in the evaluation of pericolon and periesophageal tissues; they can detect infiltration, masses, and fecalomas that cannot be detected by conventional radiography.

Endoscopy is not essential in the diagnosis of esophageal or colic involvement but is useful in assessing the state of inflammation of the mucosa and excluding the presence of cancer. While in the presence of symptoms, the above examinations should be done, there is no agreement in the case of asymptomatic patients.

Resting ECG and chest X-ray are mandatory in all patients with positive *T. cruzi* serology to detect conduction defects and dilated cardiomyopathy. In our clinical practice, all patients, even asymptomatic ones, also undergo echocardiogram and investigations of the gastrointestinal tract (esophagus X-ray and opaque enema). The 24 h Holter ECG and further cardiological or gastroenterological investigations (cardiac MRI, endoscopy, manometry) are generally reserved as indicated by a specialist.

**Figure 2:**
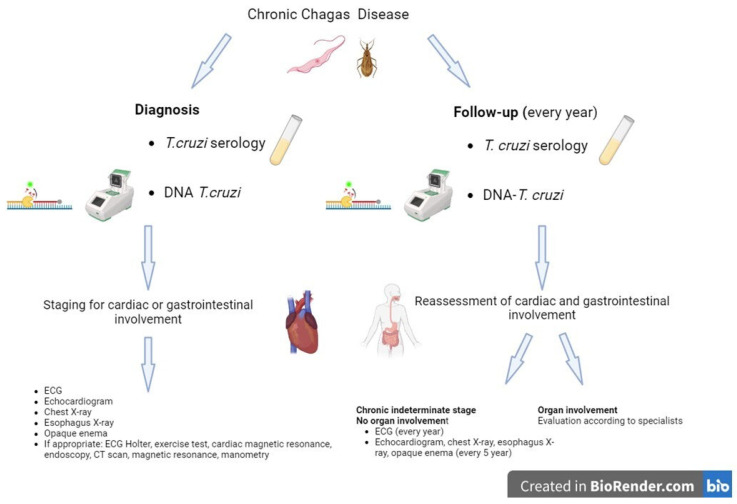
Flow chart for the clinical management of patients with Chagas disease (created in BioRender.com (BioRender, Toronto, Canada).

Figure 2 (created by BioRender.com (BioRender, Toronto, Canada)) shows the flow chart for the clinical management of patients with CD.

Organ damage should be treated in cooperation with specialists.

Asymptomatic patients should be annually reassessed by medical examination and ECG. In addition, every five years, it is useful that patients with no evidence of alterations also undergo instrumental examinations as restaging. Follow-up should continue throughout the patient’s life [36,37].

#### 1.1.7. Therapy

Benznidazole and nifurtimox have a proven efficacy against *T. cruzi*. They are the only two antiparasitic drugs approved for the treatment of CD, but their use is burdened by a high rate of side effects. Of the two drugs, benznidazole is the most widely used—preferred because of its better tolerability profile—although a high percentage of patients develop an adverse reaction, especially involving the skin [38]. Rash is frequent but commonly mild and can be managed with antihistamines and, if necessary, steroid therapy without stopping treatment. Neuropathy, leucopenia, and liver toxicity are less frequently observed. 

The dosage of benznidazole is 5 mg/kg per day (maximum dose 300 mg/day, generally divided into two doses), and therapy lasts 60 days. 

Adult dosage of nifurtimox is 8–10 mg/kg per day divided into four daily doses for 90–120 days. Adverse effects include abdominal pain, nausea and vomiting, diarrhea, anorexia, weight loss, irritability, anxiety, insomnia, depression, dizziness, tremors, myalgia, arthralgia, fatigue, fever, skin rash, itching. 

Neither drug can be used during pregnancy.

Treatment of CD is recommended in all acute and congenital cases and in cases of reactivation in immunocompromised persons [21]. Therapy is also recommended for young people and for women of childbearing age because of the proven efficacy in preventing vertical transmission [21]. Treatment is generally offered to patients with chronic CD, although the effectiveness of treatment in adults is controversial [21,38,39]. Moreover, the BENEFIT trial demonstrated that treatment with benznidazole did not significantly reduce cardiac clinical impairment when used in patients presenting moderate and severe cardiomyopathy [40]. No randomized, placebo-controlled trial on effectiveness of benznidazole for prevention of progression to cardiomyopathy in the indeterminate stage of chronic CD is available.

Early identification of patients with chronic CD who will later develop organ damage is currently impossible, and this leads to “overtreatment” for those patients (around 60% of those infected) who would never have developed complications in their lifetime. Moreover, treatment is often offered to asymptomatic persons, which makes acceptance more difficult for the patient. On the other hand, when complications arise and organ damage has set in late stage of heart and gastrointestinal involvement, CD may no longer be responsive to treatment, as the mechanism of damage is sustained by fibrotic process than by the parasite itself. However, the response to antiparasitic treatment is a condition for a possible clinical improvement and for slowing down the evolution of the disease. Negative PCR for *T. cruzi*, in cases where it is positive prior to the treatment, is a useful marker of clinical response. 

Different treatment schedules are being studied to shorten the duration of therapy and reduce side effects [41,42], as well as studies on different molecules are evaluating their efficacy in CD.

To date, there are no markers of clinical response or tests of cure.

## 2. Materials and Methods

### 2.1. Narrative Review

For the first part of this article, we summarized the evidence collected through a review of the existing literature addressing CD. Our aim was creating a brief but comprehensive overview on the disease, including its etiology, pathogenesis, epidemiology, clinical manifestations, diagnosis, screening, staging and therapy, particularly focusing on clinical management in non-endemic countries. 

### 2.2. Personal Experience on the Spallanzani Cohort

We reported the results of our experience at the National Institute for Infectious Diseases “Lazzaro Spallanzani”, IRCCS, in Rome on screening, diagnosis, and treatment of CD from 2014 to the present. Demographic, clinical, and radiological data of the patients were obtained retrospectively from the medical records. All the patients included in the analysis signed informed consent. The studies were approved by the ethics committee of our institute. 

## 3. Results

### Personal Experience on Screening, Diagnosis, and Treatment of CD at Spallanzani Institute

To date, a total of 47 CD patients have received therapy for CD. Once CD had been identified and diagnosed, all patients underwent staging to investigate potential cardiac or gastrointestinal complications and were offered treatment according to international recommendation. The standardized radiological evaluation included chest X-ray, esophagus X-ray and opaque enema, ECG and echocardiogram and, in selected cases, Holter ECG, EGDS, and colonoscopy were also performed. Cardiac MRI was performed only in one patient with acute cardiac involvement. Abdominal ultrasound was also performed to exclude other disorders. 

As mentioned, most of the patients were identified from the 2014 screening [15], and others later tested positive in our center or were referred from other hospitals. Some of the screening-positive patients in 2014 were lost to follow-up and were not treated. Table 1 shows the main characteristics of the patients who received treatment. 

Of the treated patients, the majority were female (37, 79%), with a median age of 51 years at the time of treatment (IQR 43–55). Most patients were from Bolivia (33 patients); 1 patient was Italian nationality who had a long volunteer stay in Guatemala. This latter patient was 69 years old and presented with an acute infection with myocarditis (clinical and MRI picture) and positive PCR for *T. cruzi* in his blood. He received benznidazole and cardiological therapies and recovered. All other treated patients had a chronic CD infection. 

All the patients received benznidazole 5 mg/kg (maximum dose 300 mg/day, divided into two doses), mostly provided by the WHO, in combination with B-complex vitamins and cetirizine, an antihistamine drug, to reduce the risk of side effects.

Only one patient, not immunosuppressed, received treatment twice due to reactivation of *T. cruzi* identified by positive PCR: the first time in 2014 with benznidazole for 60 days and the second time in 2023 with nifurtimox, which was stopped after 14 days due to adverse events.

Staging revealed that 34 out of the 46 patients (74%) had chronic CD without organ involvement (Table 1). Six patients had gastrointestinal involvement (non-severe esophagopathy or dolichosigma), with three also having cardiomegaly. A total of nine patients had cardiac involvement, with two treated using antiarrhythmic drugs (one presented also cardiomegaly) and two, although young, requiring pacemaker implantation; the remaining patients presented grade I or II cardiomegaly upon chest X-ray. All 10 patients with positive PCR at diagnosis were negative after treatment. While taking the drug, patients underwent regular medical examinations and blood tests every 7–15 days to check blood cells count and liver and kidney function. The treatment was well tolerated by 35 patients (74%) (Table 1). However, a high proportion of patients, 26% (12 patients) had adverse reactions to benznidazole, in agreement with the data reported in the literature [43]. Adverse reactions included skin toxicity in all observed cases; three patients also showed liver toxicity with a slight elevation of transaminases in two cases. In one patient, severe hepatotoxicity occurred after 30 days of treatment, with the histological finding of drug-induced liver injury (DILI), which was successfully resolved with high-dose steroid therapy and has already been described elsewhere [44]. Therapy had to be discontinued in 8 patients (17%) due to severe side effects; only two discontinued within the first 15 days, while 5 patients continued treatment beyond 40 days and can be considered sufficiently treated. Notably, 4 patients continued therapy for the full 60 days despite adverse skin reactions with the addition of steroid therapy to manage the side effects. The two patients who stopped treatment early did not make a further attempt with nifurtimox due to the drug’s greater toxicity and the patient’s unwillingness to retreat.

Indeed, our experience in CD management at the National Institute for Infectious Diseases “Lazzaro Spallanzani” in Rome started 10 years ago. The first extensive screening program organized by the Institute in collaboration with the international organization Médecins Sans Frontières (MSF) was carried out, and the care of CD patients began [15]. As already reported, a screening campaign for anti-*T. cruzi* antibodies targeting migrants from Latin American countries residing in Rome and the Latium Region (Italy) was promoted from February to June 2014. The potential target amounted to 37,197 people in 2014 according to an official estimate. The project was planned and implemented through a community approach to raise awareness of health promotion issues, with the support of cultural mediators, Latin American embassies, consulates, and cultural associations. Participation in the screening was voluntary, open and free of charge. During the five months of screening, 368 people from Latin America participated. Of these, 264 (71.7%) were women and 104 (28.3%) were men. The median age of the study population was 42 years (IQR: 33–51 years). Regarding the geographical distribution of participants, 115 individuals (31.2%) were from Bolivia, 123 (33.4%) from Ecuador, 62 (16.8%) from Peru, 28 (7.6%) from Colombia, and 20 (5.4%) from other Latin American countries. Additionally, 20 individuals (5.4%) were born in Italy but were included because their mothers were women of Latin American origin or Italian citizens with a history of long-term residence in Latin American countries. The tests used were: (i) the lysate-antigen-based ELISA (BioELISA Chagas III, BiosChile, Santiago, Chile), (ii) the chemiluminescent microparticle immunoassay (CMIA, Architect Chagas^®^, Abbott Diagnostics, Abbott Park, IL, USA), (iii) an immunochromatographic (ICT) assay, (Chagas Quick Test ICT, Cypress Diagnostics, Langdorp, Belgium), used in case of discordant results. As previously reported, of 368 participants, 32 (8.7% of the tested population) were found positive for *T. cruzi* antibodies, mostly from Bolivia. Higher age, birth in Bolivia, and previous residence in a mud house were independent factors associated with positive serology for *T. cruzi* in our study [15], similar to results reported in the literature [11].

In addition to the patients identified in the screenings at our center in 2014 and thereafter, eight subjects were referred to our attention because they tested positive in blood donation screening or in other hospitals.

Although activities to reach the target population have continued to date through educational programs and information at obstetrical centers, Latin American embassies, and communities and through open and free days dedicated to screening in addition to the routine outpatient examinations and tests. Nevertheless, a large part of the target population in Rome remains untested. Further screening campaigns, also organized via cultural mediators, are therefore necessary in order to reach a larger proportion of the susceptible population.

A particular population of attention are HIV-infected patients from Latin American countries, as immunosuppression can cause reactivation and result in severe disease. In an Italian multicentric study—to which our institute contributed by providing anonymized records and plasma of HIV cohort added to all national cases recorded—aimed at assessing the seroprevalence of Chagas disease in HIV-infected patients naive to antiretroviral therapy (ICONA cohort) originating from Latin America enrolled from 1997 to 2018, out of 389 patients, 15 (3.86%) had at least one positive test for Chagas, and the prevalence of Chagas disease in this particular population was found to be 0.5%–1.29% depending on the confirmation method used [45]. Screening for Chagas disease was performed using two commercial ELISA seroassays: one based on recombinant antigens (BioELISA Chagas, Biokit, Lliça d’Amunt, Spain), and one on *T. cruzi* lysate antigenic preparation (BioELISA Chagas III, BiosChile, Santiago, Chile). In case of discordance, an immunoblotting assay based on *T. cruzi* extract (CHAGAS Western Blot IgG, LDBio Diagnostic, Lyon, France) was performed. None of the HIV-positive subjects of the study were part of the case series of CD patients treated at our centre and reported above.

## 4. Discussion

While CD is a rare disease in non-endemic countries, its global burden is high. A few years ago, the global annual burden of CD was estimated at USD 627.5 million in healthcare costs and 806,170 disability-adjusted life-years (DALYs), with 10% of this burden in non-endemic countries [46]. In recent years, the global prevalence of CD has decreased by 11.3% from an estimated 7,292,889 cases in 1990 to 6,469,283 in 2019. Over the same period, the global rate of DALY from CD also decreased by 23.7%. However, the prevalence has followed different trends depending on the region, with a sustained decrease in Latin America and an increasing trend in North America and Europe [47]. Therefore, despite most cases of CD being concentrated in Latin America, the increase observed in North American and European countries highlights the importance of screening at-risk populations and raising public and medical awareness of this neglected tropical disease. In fact, the number of people affected and the burden of related morbidity and mortality are not exactly estimated. Therefore, it is necessary for clinical institutions to systematically, routinely, and actively offer CD testing to all persons presenting an epidemiological risk, such as birth, migration or adoption from an endemic country, or travel to endemic areas. Special attention should be paid to children born to mothers from Latin American countries and to women of childbearing age.

In the management of CD in non-endemic countries, many difficulties in assessment and clinical management are faced. Firstly, as already mentioned, the lack of knowledge about this neglected disease among patients and the limited medical experience of physicians are the main limiting factors. 

In addition, there are other difficulties in diagnostic and therapeutic management, such as the relative difficulty in accessing the health care system, the difficulty of finding drugs for treatment and keeping patients in follow-up. Suarez and other authors have elaborated a strategy for CD in non-endemic countries consisting of several points, which can be inspiring [37].

There are still many unclear aspects of CD, particularly the predisposing factors to the infection (i.e., why some individuals, for example within the same family, become infected and others do not), the evolution of the disease and the development of organ damage (why some people develop organ damage and most people do not), how it is possible to detect it early on, and the occurrence of toxicity during therapy. These unsolved questions need to be addressed in order to achieve a personalized approach to patients.

In our experience, we have observed that there is a high number of participants when a screening campaign is conducted with easy access to the health facility and with the support of cultural mediators and the community involvement, as in our first experience in 2014, when 368 people voluntarily underwent the serological test for diagnosis.

The patients affected by CD followed at our center are mainly from Bolivia; indeed, a large percentage of Bolivian immigrants reside in Italy and Bolivia is one of the countries with the highest prevalence of CD in Latin American. In our case series, women are more represented, and this is probably due to a greater willingness to access health care facilities than men, as there is no evidence of a greater impact of CD in women [47].

Another issue to note is that the median age of the patients treated at our center is 51 years, whereas, as mentioned above, it is recommended to administer the treatment to younger people who may have the most benefit. After the age of 50, the offer of CD therapy is optional, according to the US guidelines.

In addition, about a quarter of our patients (26%) had cardiac or gastrointestinal involvement, or both, highlighting the need for multidisciplinary management of patients.

As is well known, benznidazole treatment is burdened by numerous side effects. In our case series, we observed benznidazole toxicity in a high percentage of patients (26%), consistent with data from the literature, although this resulted in early discontinuation of therapy in only two patients, and the patients often were able to complete the treatment by associating steroid therapy. Skin reaction was present in all patients who experienced toxicity to benznidazole. One patient presented severe drug-related hepatitis (DILI) [44].

We also observed in our case series an Italian patient affected by acute CD with myocarditis who had spent a long period of volunteer activities in Guatemala. This case emphasizes the importance of suspecting and treating CD early by carefully identifying the risk factors and clinical picture of this illness.

Our experience, like other similar experiences in Europe [48], underlines the need for a structural and continuous screening program, especially in obstetric settings, to stop vertical transmission, as is already the case for blood banks and organ transplants in Italy. The treatment of affected patients with the available drugs shows significant problems, as also evidenced by our experience, and prompts the implementation of better tolerated drugs or shorter treatments and adverse event management strategies. 

## 5. Conclusions

Epidemiological data and our personal experience showed that CD is a neglected tropical disease that a physician could also face in their clinical practice in a non-endemic country. It is very important to recognize this disorder and its complications and address the patients to referral centers. Continuous screening campaigns targeted at populations at risk is mandatory for identifying asymptomatic patients, for treating them early, and for achieving control of transmission in non-endemic countries. Furthermore, it is imperative to prioritize investment on this NTD.

## Figures and Tables

**Table 1:** **Study patients.** Main characteristics of the 47 patients affected by Chagas disease treated with benznidazole. N = number; CD = Chagas disease; PCR = polymerase chain reaction; DILI = drug-induced liver injury.

Patients (N)	47
Gender (N, %)	
-Female	37 (79%)
-Male	10 (21%)
Age (years, median)	51 (IQR 43–55)
Country of origin:	
-Bolivia	33 (70.2%)
-El Salvador	5 (10.6%)
-Honduras	3 (6.4%)
-Brazil	3 (6.4%)
-Argentina	1 (2.1%)
-Colombia	1 (2.1%)
-Italy (country of infection: Guatemala)	1 (2.1%)
Concordant two tests for *T. cruzi* serology	46 (97.9%)
Positive *T. cruzi*-DNA by PCR at diagnosis	10 (21%)
Stage of CD:	
-Acute (myocarditis)	1 (2.1%)
-Chronic	46 (97.9%)
-Chronic indeterminate	34 (74%)
-Cardiac involvement	6 (13%)
-Gastrointestinal involvement	3 (6.5%)
-Cardiac and gastrointestinal involvement	3 (6.5%)
Benznidazole treatment	47 (100%)
Adverse effects to benznidazole	
-None	35 (74%)
-Any	12 (26%)
-Cutaneous	12 (26%)
-Hepatic	3 (6.4%)
-DILI	1 (2.1%)
Treatment discontinuation	
-Any	8 (17%)
-Before 40 days	2 (4.3%)
-After 40 days	5 (10.6%)
Use of steroids associated with benznidazole	10 (21%)

## Data Availability

The raw data supporting the conclusions of this article will be made available by the authors on request.
